# The use of Guyton’s approach to the control of cardiac output for clinical fluid management

**DOI:** 10.1186/s13613-024-01316-z

**Published:** 2024-07-04

**Authors:** Sheldon Magder

**Affiliations:** McGill University Health Centre, 1001 Decarie Blvd, H4A 3J1 Montreal, QC Canada

**Keywords:** Vascular volume, Stressed volume, Venous return, Right ventricle, Means systemic filling pressure, Right atrial pressure, Central venous pressure

## Abstract

Infusion of fluids is one of the most common medical acts when resuscitating critically ill patients. However, fluids most often are given without consideration of how fluid infusion can actually improve tissue perfusion. Arthur Guyton’s analysis of the circulation was based on how cardiac output is determined by the interaction of the factors determining the return of blood to the heart, i.e. venous return, and the factors that determine the output from the heart, i.e. pump function. His theoretical approach can be used to understand what fluids can and cannot do. In his graphical analysis, right atrial pressure (RAP) is at the center of this interaction and thus indicates the status of these two functions. Accordingly, trends in RAP and cardiac output (or a surrogate of cardiac output) can provide important guides for the cause of a hemodynamic deterioration, the potential role of fluids, the limits of their use, and when the fluid is given, the response to therapeutic interventions. Use of the trends in these values provide a physiologically grounded approach to clinical fluid management.

Primary roles of the circulation are to deliver oxygen (O_2_) and nutrients to tissues and to remove wastes. Delivery of O_2_ (DO_2_) is based on three factors: cardiac output, hemoglobin concentration, and the saturation of hemoglobin with O_2_ saturation. These are the only three factors that an intensivist can manipulate to improve DO_2_. The range of possible changes in hemoglobin concentration and O_2_ saturation are normally small and thus changes in cardiac output dominate the regulation of DO_2_. Arthur Guyton argued that cardiac output is determined by the interaction of two functions: (1) a function that determines the return of blood from the peripheral circulation, that is, venous return; and (2) a function that determines the output from the heart acting as a pump. Vascular volume is central to both functions. An understanding of how cardiac output is regulated by the interaction of these two functions provides a better understanding of what fluid therapy can and cannot do, and why normal values are what they are [1, 2].

## Venous return function

Guyton’s approach starts with the concept of mean circulatory filling pressure (MCFP) [3]. The volume contained by the circulation stretches the elastic walls of vascular compartments and creates an elastic recoil pressure. Because of the existence of this recoil force, when a vessel is punctured volume flows out of the vasculature even without a pumping heart. Volume filling the vasculature is made up of two components. One, unstressed volume, just rounds out vessel walls, but does not stretch vascular walls, and does not create pressure. The second, stressed volume, stretches vessel walls and creates the vasculature pressures [4]. Under basal conditions, stressed volume is about 30% of total blood volume, which is about 1.3 to 1.4 L in a 70–75 kg male [5]. MCFP is determined by total stressed volume divided by the sum of the compliances of all vascular structures, including the pulmonary and cardiac volumes. Typical MCFP in a resting normal person is in the range of 7 to 10 mmHg. Generation of blood flow by the heart occurs when the right heart empties the volume it contains and thereby lowers right atrial pressure (RAP). Except in rare conditions, RAP and central venous pressure (CVP) are the same; we will use RAP. The decreased downstream pressure allows venous blood to flow back to the heart in a cyclic manner. The heart thus has a “permissive” role in that it allows upstream venous volume to flow back to the heart. Since the venous compartment upstream from the heart is the most compliant part of the vasculature and contains most of vascular volume [6], the pressure in this region changes very little during the cardiac cycle and is usually very close to MCFP [7]. . However, the venous pressure in this region can differ from MCFP depending upon the distribution of flow. Because of the key importance the upstream pressure in the systemic veins for venous return of, this pressure is given its own name, mean *systemic* filling pressure (MSFP). The final factor determining flow from systemic veins back to the right heart is the effective resistance in the veins (RVR) between MSFP and the right heart. Venous return function (VR) thus can be summarized as:

1 $$\text{V}\text{R} = (\text{M}\text{S}\text{F}\text{P}-\text{R}\text{A}\text{P})/\text{R}\text{V}\text{R}$$

**Fig. 1:**
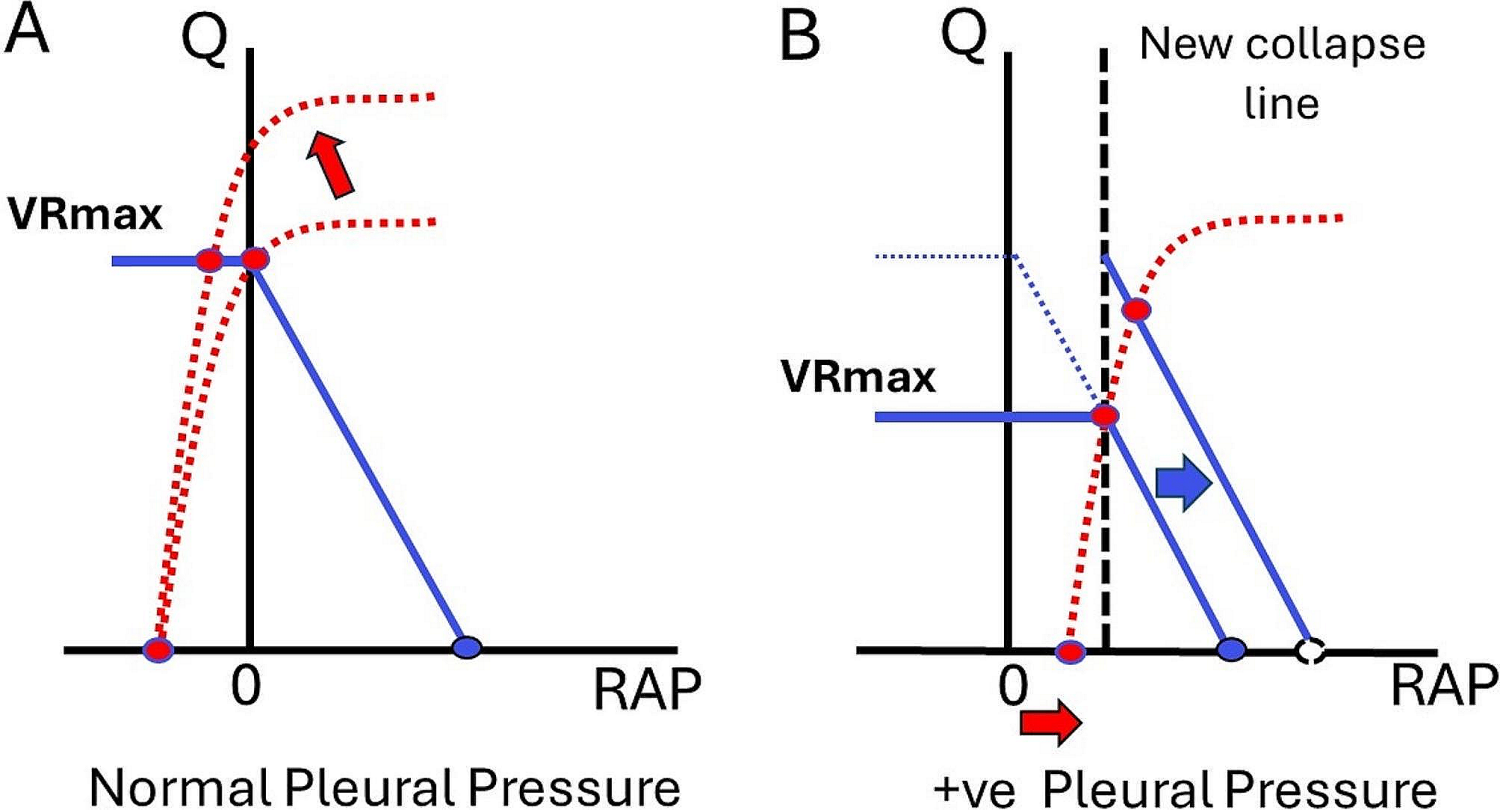
**Limitations to venous return and maximum VR (Vmax) with normal pleural pressure (A) and Positive pleural pressure (B).** In A, when VR intersects the 0 line (atmospheric pressure) venous collapse occurs. A increase in the cardiac curve (dotted line) in this state does not increase cardiac output (closed red circle). In B, a rise in pleural pressure results in venous collapse and flow limitation at a pressure above atmospheric pressure. A shift of the venous return curve to the right is then needed to increase VRmax. This can occur by giving volume or by conversion of unstressed into stressed volume through reflex mechanisms or drugs (see text). RAP is right atrial pressure and Q represents cardiac output.

There is an important limit to venous return [8]. When the pressure outside the great vessels returning blood to the heart is greater than the pressure inside these vessels, they collapse. This creates what is called a vascular waterfall or flow limitation [9] (Fig. 1). The collapse very transiently blocks the vessel and stops flow, but the pressure inside the vessel quickly starts to rise to the upstream pressure, which transiently re-opens the vessel. The pressure in the vessel then flutters with opening and closing at the surrounding pressure creating the collapse. When a person is breathing spontaneously and is upright, the RAP fluctuates around zero (relative to atmosphere). However, during mechanical ventilation, venous collapse pressure occurs at a positive pleural pressure relative to atmosphere. The implication of the venous collapse point is that the best the heart can do is lower RAP to zero, i.e. atmosphere, when breathing spontaneously, and a positive value when mechanically ventilated. Thus, maximum cardiac output is determined by circuit factors and not the heart. A RAP greater than zero just reduces VR. Maximum VR is then determined by:

2 $$\text{V}\text{R}\text{m}\text{a}\text{x} = \text{M}\text{S}\text{F}\text{P}/\text{R}\text{V}\text{R}$$

When VR is limited by the collapse, cardiac output only can be increased by increasing stressed volume and thereby increasing MSFP.

Although increasing MSFP can potentially increase cardiac output, there is a price to pay. The typical pressure difference from MSFP to RAP is 3 to 8 mmHg [10, 11]. If RAP is 10 mmHg, and assuming a pressure difference of 6 mmHg, MSFP would be 16 mmHg. The pressure difference from MSFP to the venous side of the capillaries is ∼ 10 mmHg. This would then give a capillary venous pressure of 26 mmHg. The pressure drop across capillaries is ∼ 10 mmHg [12]. Accordingly, the capillary pressure on the arterial side would start at 36 mmHg. At this high pressure, capillary filtration would be greatly increased. If capillary permeability also is increased because of inflammation, or if oncotic pressure is decreased because of decreased serum albumin, filtration will increase even more. It thus should be evident that a RAP much greater than 10 mmHg should be used cautiously and it should make the physician re-consider the necessity of giving more volume, even if the patient is volume responsive.

## Change in the return function

The primary way that VR can be increased is by giving volume and increasing MSFP. The body can do this by recruiting unstressed volume into stressed volume through a baroreceptor mediated mechanism and by vasoconstrictors such a alpha receptors agonistes [13], angiotensin [14], endothelin-1 [15], and neuropeptide Y [16]. This acts as an “auto-transfusion” [17]. A decrease in venous resistance also increases venous return. This occurs during exercise [18] likely through flow medicated dilation as well distension by increase vascular pressures, as well as with the use of inotropic drugs such as dobutamine, milrinone and perhaps even norepinephrine through their binding to beta receptors which actively dilate venous resistance vessel [19, 20]. In contrast, although phenylephrine can recruit unstressed volume, it increases venous resistance and so almost always decreases venous return and cardiac output [21, 22]. Finally, the most important factor that can increase venous return is a decrease in RAP by an increase in cardiac function. It is noteworthy that given the normal pressure difference of MSFP to RAP of 4 to 6 mmHg, a change of RAP of 2 to 3 mmHg either up or down by what ever process, would change venous return and cardiac output by 50% if there are no other circuit adjustments. It is thus of paramount importance that RAP is measured precisely.

## Cardiac function

The second component is cardiac function as described by Ernest Starling [23, 24]. Starling’s cardiac function curve indicates that the greater the stretch of cardiac muscle during diastole, the greater the force generated by the heart, and the greater the stroke output at a constant afterload, contractility, and heart rate. This is true up to a maximum diastolic volume at which diastolic filling is limited by the pericardium, or by the cytoskeleton of the walls of the heart [25]. This filling limit creates a sharp flatting of the cardiac function curve. The break is much sharper than drawn in many texts and articles. When the break is reached, infusing more volume will increase RAP but will not change RV diastolic volume and thus the adding volume will not increase SV. When RV filling is limited, excessively increasing diastolic pressure can even depress cardiac function by hindering coronary prefusion. Cardiac function, that is, stroke output for a given preload, can be increased by decreasing ventricular afterload, increasing contractility, or by increasing heart rate (allows more stroke volumes per minute). It is worth emphasizing that the RV sets maximum SV [26]. This is because the left heart only can put out what the right heart gives it. When the left heart cannot handle the volume it gets, the increase in left sided pressure raises the load on the RV. This increases RV diastolic volume, which eventually becomes limited. On the other hand, the left heart determines the arterial pressure for distribution of flow to organs. If the left heart fails to handle the output from the right heart, pulmonary edema rapidly develops.

## Guyton’s graphical analysis

**Fig. 2:**
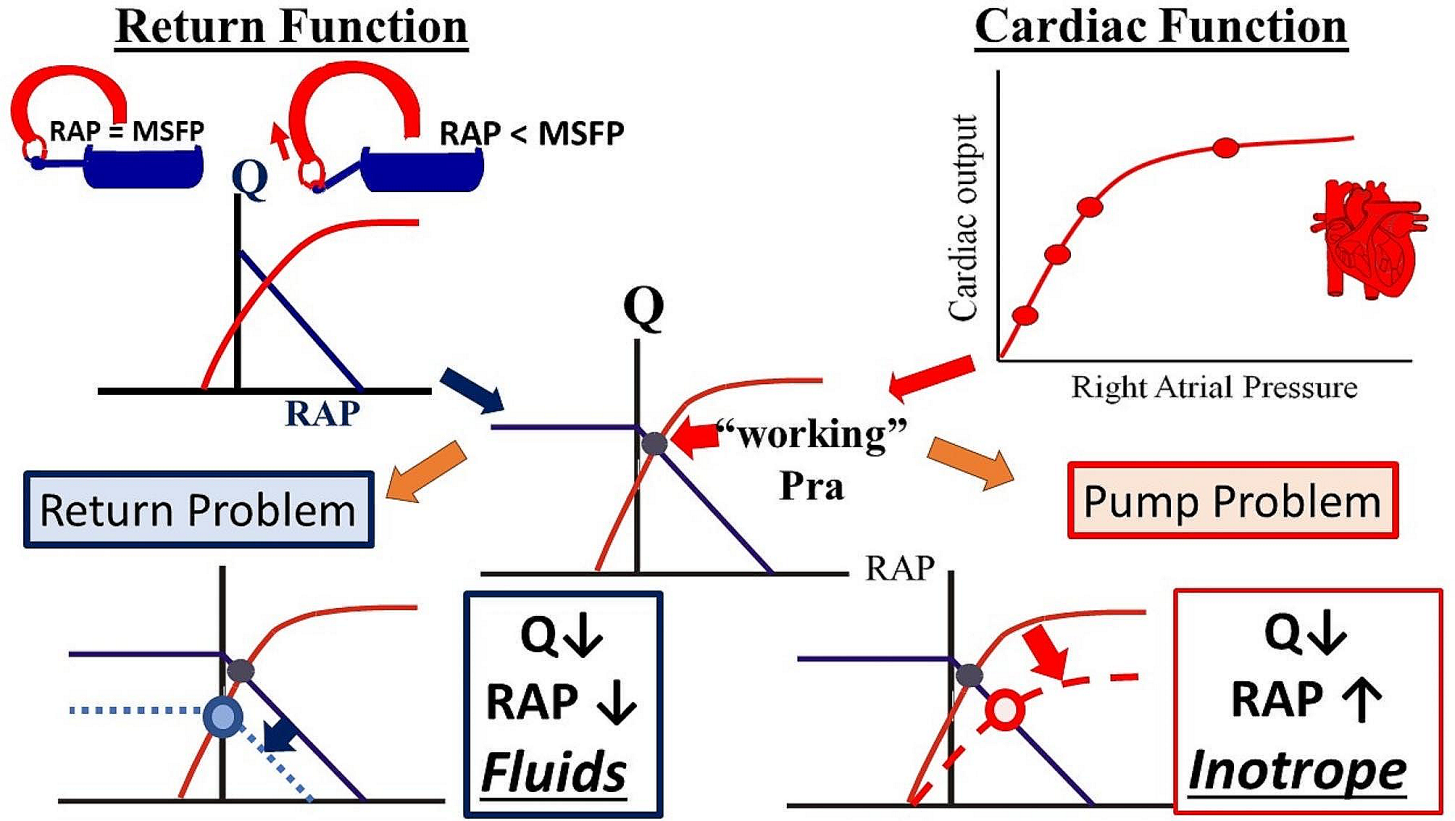
**Control of cardiac output by the interaction of the venous return function (left upper) and cardiac function (right upper).** When RAP = MSFP there is no flow. Lowering RAP (by the pumping heart), allows for venous return to occur. The intersection of the cardiac function and return function (centre) gives the “working” right atrial pressure (RAP), “working” cardiac output and “working” venous return. “Q” is flow. See text for further details. Blue lines indicate VR and red line cardiac function. A decrease in cardiac output (Q) with a fall in RAP (left lower)indicates a primarily “return” problem. Fluids are likely the best choice. A decrease in Q with an increase in RAP (right lower) indicates a primarily “Pump” problem. An inotrope is likely the best clinical choice. See text for further details.

Since both VR and cardiac functions have the same axis, they can be plotted together (Fig. 2). The RAP at which the two functions intercept indicates the functioning preload of the RV, the back pressure for VR, and the cardiac output. If cardiac function increases, the heart effectively becomes “more permissive”. Cardiac output increases and RAP falls so that cardiac output and RAP change in opposite directions. When cardiac function decreases, cardiac output falls and RAP rises. In contrast, when VR function increases, cardiac output rises and so does RAP and when VR function falls cardiac output falls and so does RAP, i.e. the changes are in the same direction.

## Clinical usefulness of the principles in the Guyton plot

An understanding Guyton’s cardiac-venous return plot can be very useful clinically, especially for volume management (Fig. 2).

### Use of the magnitude of RAP

To begin, the magnitude of RAP has a clinical utility in and of itself that often is not appreciated [27]. Importantly, RAP even can be evaluated as being high or low in many patients without any sophisticated technology by just observing jugular venous distention. The RAP value gives an indication of how well the heart is doing compared to volume coming back to it. However, an noted by Swan and Ganz, it does not tell you that the failing LV is producing to pulmonary congestion [28]. It is noteworthy that Guyton called the cardiac function curve a “cardiac response curve”. When the heart puts out more than is coming back, RAP goes to zero and tVR then sets the limit to cardiac output. In thi situation, only adding volume can increase cardiac output. This is very evident when the circuit has a mechanical heart. When blood does not come back fast enough, the venous outflow drains start chattering and perfusionist ask for more volume to be given. The added volume shifts the VR curve to the right and VR intercepts the y-axis (flow) at a higher plateau value (i.e. maximal flow). It is worth noting that not a lot of volume is necessary to test this. On the other hand, a high RAP indicates that the heart as a whole is not adequately handling the returning flow; this can be due to either RV or LV dysfunctiion. When the RAP is much above 10 mmHg, the failure of the heart to keep it lower leads to organ congestion [27]. The kidney and liver are especially vulnerable. A high RAP also indicates that the heart is either on the flat part of its function curve, or very close to the plateau, and thus more volume will not increase cardiac output. The volume given also will leak out of the vasculature more quickly. Non-volume therapies should then be considered such as vasopressors for blood pressure and inotropes to increase cardiac function.

### Diagnostic use of changes in RAP

It is worth starting with another simple relationship. Except for a small downstream pressure, blood pressure is approximately equal to the product of cardiac output and systemic vascular resistance (SVR). The implication is that a fall in blood pressure is due to either a fall in cardiac output or a fall in SVR. Furthermore, what is measured is blood pressure and cardiac output, or at least clinical surrogates of cardiac output. However, SVR is calculated from the other two variables. Thus, to determine which factor has caused the fall in blood pressure, it first needs to be asked: is cardiac output normal or elevated? If so, a decrease in SVR is the primary problem. Increasing cardiac output might still restore arterial pressure, but if the pressure drop was severe, the resistance needs to be increased with a vasopressor. If cardiac output is decreased, then the low cardiac output is the major problem. As per Guyton’s analysis, cardiac output could be decreased because of depressed pump function or because of reduced venous return. Which of these is the dominant problem can be distinguished by examining the change in RAP at the intersection value of the two curves. If the RAP at the intersection is low, depressed return is the most likely process, whereas if RAP is elevated, decreased cardiac function is the most likely process and a volume infusion is unlikely to help. An actual measurement of cardiac output is ideal for this analysis, but surrogates and their trends are often adequate. Surrogates include central venous saturation, lactate concentration, skin temperature and skin perfusion [29]. One of the best tests of adequate perfusion is the patient’s state of alertness. It only is necessary to identify the direction of changes and trends, for they identify the primary process and the response to the chosen therapy. When venous return is the problem, volume therapy is the first choice, although recruitment of unstressed volume into stressed volume by norepinephrine can help too.

### Assessment of therapy response

Another way that Guyton’s analysis can be helpful is monitoring the response to a therapy. If inadequate venous return was deemed to be the likely problem, giving volume should result in an increase in cardiac output and a rise in RAP. If on the other hand, the pump is thought to be the problem, use of an inotrope should reduce RAP. Measurements of perfusion and RAP thus can help the clinician know if the chosen therapy corrected the problem or if the patient was already just getting better independent of the intervention!

### Special problem of ventilation

Mechanical ventilation creates an important problem. Preload for cardiac function is based on the pressure across the ventricular wall, which is called transmural pressure. The pressure outside cardiac chambers is pleural pressure and not atmospheric pressure, but RAP normally is measured relative to atmospheric pressure. When a person is breathing spontaneously, pleural pressure has a negative value at end-expiration. Guyton dealt with this issue by plotting the start of the cardiac function curve at a negative value. Since negative values on the x-axis of the cardiac output-RAP plot are below the plateau of the venous return curve, more negative values of RAP do not change blood flow. However, in mechanically ventilated patients, pleural pressure is a positive number and it becomes more positive on each inflation. When pleural pressure is positive, the transmural RAP is always less than the value of RAP seen on the monitor. Variations in the true transmural RAP compared to the value measured relative to atmosphere in ventilated patients could explain why some patients still respond to fluids at higher values of the standard RAP measurement [30]. However, even though the patient may respond to fluids at these higher values of RAP, there is a price to pay for pushing RAP higher. Thus, volume-responsiveness should not be the only factor for the decision to give fluids. The actual value of RAP itself should be considered because of the risk of tissue congestion.

### Role of volume in “reserves”

A potential value for giving an initial volume bolus to a patient in shock is not covered in Guyton’s analysis. Unstressed and interstitial volumes provide important reserves that allow the body to regulate the appropriate stressed volume and appropriate MSFP through neuro-humeral processes. If volume reserves were already recruited to replenish volume losses, important normal homeostatic mechanisms have been lost. In these cases, an initial 1 to 2 L of crystalloid solution may allow the body to use its own normal homeostatic mechanism to regulate stressed vascular volume. The benefit of increasing volume reserves occurs without measurable changes in RAP, blood pressure or cardiac output and are only apparent when the system is challenged. There is no clinical measurement that can identify reduced volume reserves; these reserves only can be estimated by considering a patient’s volume history of intake and losses.

## Conclusion

Guyton’s analysis puts the RAP at the central place of the intersection of the VR function and cardiac function. As such, RAP gives an indication of how the heart is dealing with what comes back to it. Elevations of RAP also give an indication of the risk of congestion in tissues. When there is a decrease in blood pressure due to a decrease in cardiac output, directional changes of RAP indicate whether the primary problem is likely due to a decrease in cardiac function or to a decrease in the VR function. If the problem is primarily a decrease in cardiac function, inotropic therapy is likely the best choice; if decreased VR function is deemed the primary process, a volume infusions is the most appropriate primary approach. In both situations fluids and inotropes can still be adjuncts to the management.

## Data Availability

Not applicable.

## Declarations

### Authors’ information

If any of the sections are not relevant to your manuscript, please include the heading and write ‘Not applicable’ for that section.

## References

[1] Magder S. An Approach to hemodynamic monitoring: Guyton at the beside. Crit Care. 2012;16:236–43. 23106914 10.1186/cc11395 PMC3682240

[2] Magder S. Right atrial pressure in the critically ill: how to measure, what is the value, what are the limitations. Chest. 2016. 10.1016/j.chest.2016.10.026 27815151

[3] Guyton AC, Polizo D, Armstrong GG. Mean circulatory filling pressure measured immediately after cessation of heart pumping. AmJPhysiol. 1954;179(2):261–7. 10.1152/ajplegacy.1954.179.2.261 13218155

[4] Rothe C. Venous system: physiology of the capacitance vessels. In: Shepherd JT, Abboud FM, editors. Handbook of Physiology. The Cardiovascular System. Section 2. III. Bethesda: American Physiological Society; 1983. pp. 397–452.

[5] Magder S, De Varennes B. Clinical death and the measurement of stressed vascular volume. Crit Care Med. 1998;26:1061–4. 9635656 10.1097/00003246-199806000-00028

[6] Guyton AC, Lindsey AW, Kaufman BN. Effect of mean circulatory filling pressure and other peripheral circulatory factors on cardiac output. Am J Physiol. 1955;180:463–8. 14376522 10.1152/ajplegacy.1955.180.3.463

[7] Yamamoto J, Trippodo NC, Ishise S, Frohlich ED. Total vascular pressure-volume relationship in the conscious rat. Am J Physiol. 1980;238(6):H823–8. 7386641 10.1152/ajpheart.1980.238.6.H823

[8] Guyton AC, Adkins LH. Quantitative aspects of the collapse factor in relation to venous return. AmJPhysiol. 1954;177(3):523–7. 10.1152/ajplegacy.1954.177.3.523 13158606

[9] Permutt S, Riley S. Hemodynamics of collapsible vessels with tone: the vascular waterfall. J Appl Physiol. 1963;18(5):924–32. 14063262 10.1152/jappl.1963.18.5.924

[10] Jellinek H, Krenn H, Oczenski W, Veit F, Schwarz S, Fitzgerald RD. Influence of positive airway pressure on the pressure gradient for venous return in humans. J ApplPhysiol. 2000;88(3):926–32. 10.1152/jappl.2000.88.3.926 10710387

[11] Nanas S, Magder S. Adaptations of the peripheral circulation to PEEP. Am Rev Respiratory Dis. 1992;146:688–93. 1519849 10.1164/ajrccm/146.3.688

[12] Levick JR, Michel CC. Microvascular fluid exchange and the revised Starling principle. Cardiovasc Res. 2010;87(2):198–210. 20200043 10.1093/cvr/cvq062

[13] Deschamps A, Magder S. Baroreflex control of regional capacitance and blood flow distribution with or without alpha adrenergic blockade. J Appl Physiol. 1992;263:H1755–63. 10.1152/ajpheart.1992.263.6.H1755 1362332

[14] Zhang H, Han GW, Batyuk A, Ishchenko A, White KL, Patel N, et al. Structural basis for selectivity and diversity in angiotensin II receptors. Nature. 2017;544(7650):327–32. 28379944 10.1038/nature22035 PMC5525545

[15] Notarius CF, Erice F, Stewart D, Magder S. Effect of baroreceptor activation and systemic hypotension on plasma endothelin-1 and NPY. Can J Physiol Pharmacol. 1995;73:1136–43. 8564881 10.1139/y95-162

[16] Deschamps A, Magder SA. Neuropeptide-Y decreases splanchnic vascular capacitance. FASEB J. 1991;5:A774.

[17] Rothe CF. Reflex control of veins and vascular capacitance. Physiol Rev. 1983;63(4):1281–95. 6361810 10.1152/physrev.1983.63.4.1281

[18] Magder S, Famulari G, Gariepy B. Periodicity, time constants of drainage, and the mechanical determinants of peak cardiac output during exercise. J Appl Physiol (Bethesda Md: 1985). 2019;127(6):1611–9. 10.1152/japplphysiol.00688.2018 31414960

[19] Datta P, Magder S. Hemodynamic response to norepinephrine with and without inhibition of nitric oxide synthase in porcine endotoxemia. AmJRespCritCare Med. 1999;160(6):1987–93. 10.1164/ajrccm.160.6.9808019 10588618

[20] Green JF. Mechanism of action of isoproterenol on venous return. Am J Physiol. 1977;232(2):H152–6. 842647 10.1152/ajpheart.1977.232.2.H152

[21] Magder S. Phenylephrine and tangible bias. Anesth Analgesia. 2011;113(2):211–3. 10.1213/ANE.0b013e318220406a 21788324

[22] Thiele RH, Nemergut EC, Lynch C III. The clinical implications of isolated alpha 1 adrenergic stimulation. Anesth Analgesia. 2011;113(2):297–304. 10.1213/ANE.0b013e3182120ca5 21519053

[23] Guyton AC. Determination of cardiac output by equating venous return curves with cardiac response curves. PhysiolRev. 1955;35:123–9. 10.1152/physrev.1955.35.1.123 14356924

[24] Katz AM. Ernest Henry Starling, his predecessors, and the Law of the heart. Circulation. 2002;106(23):2986–92. 12460884 10.1161/01.cir.0000040594.96123.55

[25] Katz AM, Series, Elasticity. Active state. Lenght-Tension Relationship, and Cardiac mechanics. Physiology of the heart. Second ed. New York: Raven; 1992. pp. 196–218.

[26] Magder S, Slobod D, Assanangkornchai N. Right ventricular limitation: a tale of two elastances. Am J Respir Crit Care Med. 2022. 10.1164/rccm.202106-1564SO 36257049

[27] Pesenti A, Slobod D, Magder S. The forgotten relevance of central venous pressure monitoring. Intensive Care Med. 2023;49(7):868–70. 37294343 10.1007/s00134-023-07101-z

[28] Ganz W, Donoso R, Marcus HS, Forrester JS, Swan HJC. A new technique for measurement of cardiac output by thermodilution in man. Am J Cardiol. 1971;27:392–6. 4929422 10.1016/0002-9149(71)90436-x

[29] Hernández G, Ospina-Tascón GA, Damiani LP, Estenssoro E, Dubin A, Hurtado J, et al. Effect of a Resuscitation Strategy Targeting Peripheral Perfusion Status vs serum lactate levels on 28-Day mortality among patients with septic shock: the ANDROMEDA-SHOCK Randomized Clinical Trial. JAMA. 2019;321(7):654–64. 30772908 10.1001/jama.2019.0071 PMC6439620

[30] Eskesen TG, Wetterslev M. A.Perner. Reanalysis of central venous pressure as an indicator of fluid responsiveness. Intensive Care Med. 2015. 10.1007/s00134-015-4168-4 26650057

